# Transformation Products of Emerging Pollutants Explored Using Non-Target Screening: Perspective in the Transformation Pathway and Toxicity Mechanism—A Review

**DOI:** 10.3390/toxics10020054

**Published:** 2022-01-24

**Authors:** Thodhal-Yoganandham Suman, Soo-Yeon Kim, Dong-Hyuk Yeom, Junho Jeon

**Affiliations:** 1 Department of Environmental Engineering, Changwon National University, Changwon 51140, Gyeongsangnam-do, Korea; sumancas2010@gmail.com; 2 School of Smart and Green Engineering, Changwon National University, Changwon 51140, Gyeongsangnam-do, Korea; 3 Gyeongnam Branch Institute, Korea Institute of Toxicology (KIT), Jinju-si 52834, Korea; sykim@kitox.re.kr (S.-Y.K.); dhyeom@kitox.re.kr (D.-H.Y.)

**Keywords:** environmental contamination, transformation products, non-target screening, transformation pathways, toxicity

## Abstract

The scientific community has increasingly focused on forming transformation products (TPs) from environmental organic pollutants. However, there is still a lot of discussion over how these TPs are generated and how harmful they are to living terrestrial or aquatic organisms. Potential transformation pathways, TP toxicity, and their mechanisms require more investigation. Non-target screening (NTS) via high-resolution mass spectrometry (HRMS) in model organisms to identify TPs and the formation mechanism on various organisms is the focus of this review. Furthermore, uptake, accumulation process, and potential toxicity with their detrimental consequences are summarized in various organisms. Finally, challenges and future research initiatives, such as performing NTS in a model organism, characterizing and quantifying TPs, and evaluating future toxicity studies on TPs, are also included in this review.

## 1. Introduction

Concerns about emerging pollutants (EPs) have increased in recent years due to their prevalence in the environment and the potential for deleterious effects on the environment [1,2,3,4]. Effluent discharges from industrial wastewater treatment plants (WWTPs), municipal, hospital, sewer overflow/sewer leakage, and surface runoff from agricultural or urban areas can all introduce EPs to the aquatic environment [5,6,7,8,9]. A special focus has been paid to WWTPs due to the relatively frequent release and high contribution of EPs into the environment. Raw influent and treated effluent commonly include EPs at concentrations ranging from ng/L to mg/L [10,11,12,13,14]. The socioeconomic composition of the population feeding into WWTPs impacts the concentrations and types of EPs in wastewaters. EPs in the water environment have often been accumulated in aquatic organisms and lead to alterations that endanger the sustainability of aquatic ecosystems [15,16].

According to the network of reference laboratories, research centers, and related organizations for monitoring emerging environmental substances (NORMAN), chemicals that are not covered by systematic environmental monitoring programs can be candidates for legislative management in the future due to their deleterious consequences and persistence. Water and wastewater treatment regulatory and implementation agencies assume that so-called priority pollutants account for most human health, environmental, and economic risk, even though they represent only a small proportion of the known and yet-to-be-identified chemical substances [17]. Unlike POPs designated in the Stockholm Convention, EPs, including an extensive range of substances, are discussed due to the scarcity of information on their occurrences and potential risks and thus the absence of management plan. Recent studies identified many different kinds of new pollutants, including perfluorinated compounds, artificial sweeteners, pharmaceuticals and disinfection byproducts, hormones and UV filters, and benzotriazoles siloxanes, naphthenic acids, musk fragrances, and TPs [18,19,20].

Identifying, detecting, and quantifying countless chemicals in the environment are significant hurdles that scientists and policymakers across the globe are now facing. In particular, tracking and recognizing TPs are challenging tasks, mainly due to the difficulty in predicting transformation pathways and the lack of a reference standard for orthogonal confirmation via instrumental analysis. Chromatography coupled with the mass spectrometer technique is the primary method for detecting TPs in the environment and organisms [21,22,23,24,25]. Recent advances in technology have extended the identification and quantification capability. For example, high-resolution mass spectrometry (HRMS) features good mass accuracy, high mass resolving power (greater than 50,000), high picomolar to femtomolar range sensitivity, and good isotopic abundance accuracy (3–20%) [26,27]. These merits rely on several operational factors, including scan speed, the mass range for an analyte, and ionization efficiency. For analysis of polar organic compounds, including most TPs present in trace amounts, liquid chromatography (LC) connected to HRMS such as time-of-flight (TOF) or Orbitrap type [28] has often been used. A new generation of quadrupole preceding tandem HRMS (e.g., QTOF and QExactive Orbitrap) is capable of highly selective and sensitive analysis for trace pollutants. In addition, the hybrid technique and sophisticated software processing mass spectrum data enables non-target screening (NTS, including suspect screening), which is applicable for the identification of less recognized and/or unknown substances such as TPs [28]. HRMS-based NTS has significantly contributed to a comprehensive understanding of TPs in environments.

Nevertheless, TPs’ occurrences and fate are likely insufficient to evaluate the toxicity and environmental risk. TPs are, in many cases, more polar and less hazardous than their parent compounds. Yet, the toxicity and persistence of TPs may vary widely depending on the alteration of molecular structures [24]. Non-mammalian model species have been developed over many years as an alternative to investigating harmful substances, since no toxic compounds can be studied in humans, and a limited number of mammal models are available [29]. Beneficial qualities such as a short lifespan and a lack of need for specialized care led to the selection of these non-mammalian models. The adverse effects of TPs have been studied using various models, including cell lines, earthworms, crustaceans, and fish. To better comprehend the impact of TPs on the larger organism, the findings of studies with model organisms are critical.

The primary goal of this study is to provide a summary of the current level of understanding for the fate of TPs and the ecotoxicological impacts on both terrestrial and aquatic organisms. We highlight how the advancement of precise mass equipment and computer/software tools has led to NTS, an essential component of an integrated approach to TP identification. Finally, we examine some critical features of the advances achieved in understanding the outcome and toxicity of TPs and the difficulties and possibilities of identifying whether these TPs can cause environmental issues in the future.

## 2. Transformation Products

**Figure 1:**
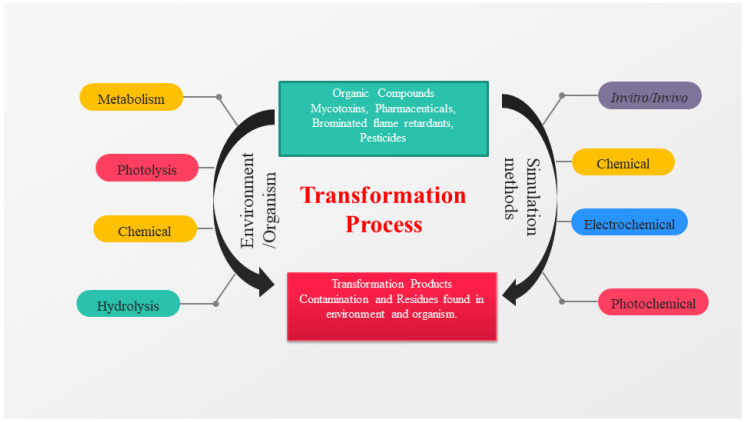
A diagram depicting the transformation processes that occur in organic compounds. General transformation processes in organisms and their environments are represented on the left, while the most frequent simulation approaches are shown on the right.

Over the previous century, the industry has generated new chemical products such as agricultural pesticides, pharmaceuticals, plastics, and dyes; unfortunately, they contain a broad spectrum of dangerous and/or persistent compounds extensively occurring in environmental compartments. The synthesized organic compounds can be categorized according to their molecular structure and functional groups (e.g., ethers, acids, alcohols, esters, hydroxyl, alkyl halides, and in each group, amines) [30]. There are a variety of abiotic and biotic processes (e.g., photolysis, hydrolysis, microbial/enzymatic metabolism, and oxidation) that might result in the formation of TPs, including the breakdown of the parent compounds (Figure 1) [31,32,33]. In the case of metabolites (or biochemically transformed products, BTPs), the parent chemical accumulated in biota is often engaged to phase 2 reactions, leading to conjugated forms, which are easily excreted due to the enhanced water solubility [34]. Some TPs are biologically active and are of concern in terms of ecotoxicity [35,36,37]. 

Unlike other compounds, emerging pollutants have less data on TPs. Specific compounds and their TPs have been identified in waste sources, such as septic systems [38,39], wastewater treatment plants [34,40,41,42], animal manure [43,44], and lands [45]. Gemfibrozil, ofloxacin, ibuprofen, irbesartan, and venlafaxine were found in wastewater treatment facilities and surface water at concentrations of more than 100 mg/L [46]. While looking for common fragments, valsartan and its three TPs were identified in waters. O-desmethyl-venlafaxine and an oxidized form of gemfibrozil TPs were detected more often than their parent compounds. Some TPs in wastewater treatment facilities were frequently detected compared to the surface waters. Ibuprofen degradation products IbB4, IbSW2, and Ib1 and the TPs of gemfibrozil, GSWB1 were the examples (Figure S1, Table 1).

According to previous studies, sulfamethoxazole TPs accounted for up to 86% of the total load in untreated wastewater [47]. Parent compounds have shown to be more persistent, with TPs accounting for 5% in treated effluent [48]. The peak area ratios of TPs in wetland were also well recorded in other research [49], with most TPs showing larger peak ratios at the exit point of the wetland compared to the inlet site. Seven TPs were eventually verified using reference standards in the study. At the same time, the identifications of the other TPs achieved high confidence levels by giving diagnostic structural evidence via fragment elucidations and MS/MS database comparisons.

**Figure 2:**
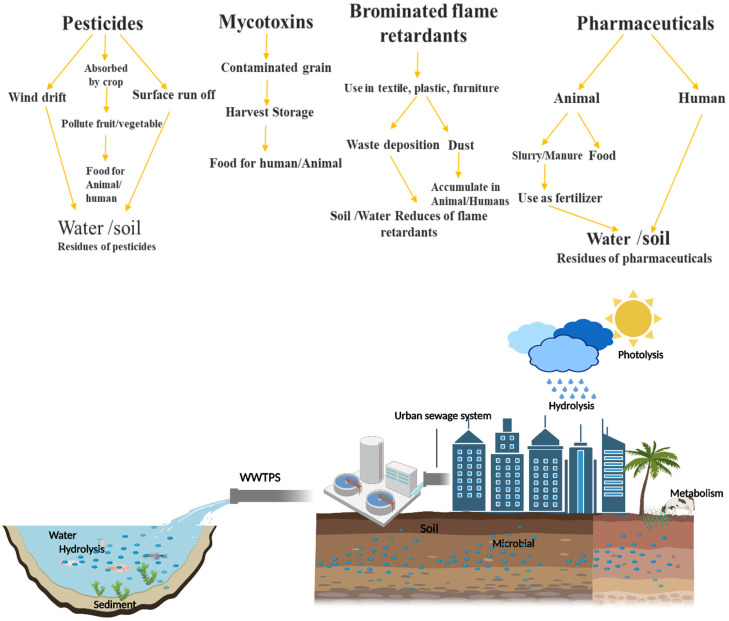
Contaminants and residues of mycotoxins, pesticides, pharmaceuticals, and brominated flame retardants may enter the environment via various routes, including water, sediment, soil, and groundwater. Hydrolysis, photolysis, metabolism, and microbial activity are the critical transformation mechanisms resulting in diverse TP formations that are broadly distributed.

Organic pollutants and their TPs are excreted and may infiltrate environmental systems (Figure 2), including groundwater, soil, sediment, and biota. These matrices might be affected by their occurrences [50,51,52]. Percussor pollutants such as pharmaceuticals [34,35], pesticides [31,32,53,54,55], surfactants [33,56,57], hormones [38,58], and personal care products [36,37] have often been found in the environment. When EPs enter the environment, they undergo biological and chemical transformations, resulting in molecular weight variations [59,60,61,62,63,64,65,66,67,68,69,70,71,72,73,74,75,76] (Table 2). Meanwhile, some TPs have been detected more significantly in the environment than their parents. Thus, the environmental prevalence of synthetic organic compounds might be significantly underestimated if TPs are not considered [77,78,79,80,81]. 

## 3. Nontarget Screening

**Figure 3:**
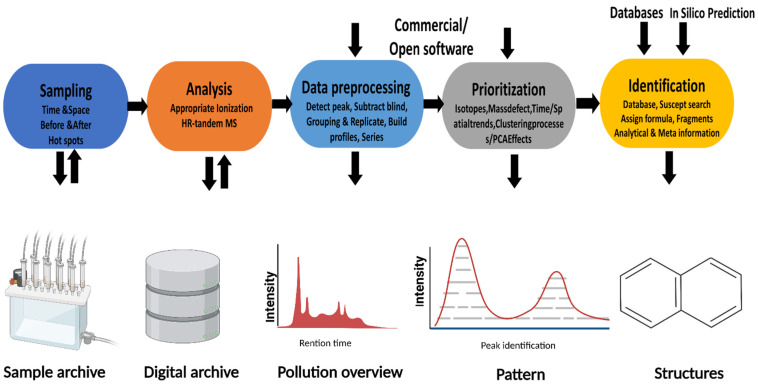
Workflow for non-target screening to identify non-targeted peaks with feasible molecular structures.

Detection of highly resolved peaks with HRMS is of the most effective ways to identify novel substances such as TPs [83]. NTS workflows were established in the early 1970s to identify unknown substances. In NTS methods, mass spectrometers and chromatograms are often used. Samples in NTS undergo an extraction procedure to retrieve analytes. Once the analytes have been identified, they may be cross-referenced with databases that provide known elements. The analyte structure would be compared to known compounds and categorized after the analyte components were found. Consequently, there would be a mixture of known and unknown chemical characteristics (Figure 3). The challenge with the HRMS tool is that the instrument generates a lot of spectrum data that should be evaluated and exported in a controlled manner. Additionally, the instrument may run in full scan and MS/MS modes simultaneously (i.e., data-dependent/independent MS/MS acquisition), generating even more data to be collected in a single run. As a post step, software-aid processing is required for the acquired spectrum data. The data processing NTS step may be performed using a variety of free and commercial software tools, including:

- XCMS (https://xcmsonline.scripps.edu accessed on: 1 December 2020);
- MZmine (http://mzmine.sourceforge.net/ accessed on: 1 December 2020);
- Non-target, ACD MS/Workbook Suite; and,
- EnviMass (http://www.eawag.ch/forschung/uchem/software/enviMass1 accessed on: 1 December 2020);
- vendors’ software, such as TraceFinder/CompoundDiscoverer (Thermo Scientific, Waltham, MA, USA), Profile-Analysis (Bruker, Billerica, MA, USA), MetaboLynx/MassLynx (Waters, Milford, MA, USA), MassHunter (Agilent, Santa Clara, CA, USA), and Data Explorer (Applied Biosystems, Waltham, MA, USA).

Choosing the best peaks is the primary step. During this phase, it is critical to compare the sample to control or blank sample to remove irrelevant peaks. Automated processes such as mass calibration, adduct componentization, and isotope peak matching are aimed at ruling out the unnecessary peaks. According to Kind and Fiehn 2007, heuristic filters describe the chemical formula to the exact mass for the hits [84].

It is possible to find potential structures via searching databases such as PubChem, ChemSpider, the NIST or structure creation, and DAIOS database. As a result, the assignment of molecular formula with substructure information can dramatically narrow down the number of candidates in databases and allow easy access to distinctive structures of the hits.

Information on MS/MS fragmentation must be compared to in silico spectral fragmentation from the library to rank the candidate structures. A few mass spectrometer databases provide MS/MS spectrum data, such as:

- MetLin (http://metlin.scripps.edu/index.php accessed on: 1 December 2020).
- MassBank (http://massbank.ufz.de/MassBank/ accessed on: 1 December 2020); mzCloud (Thermo)

To date, TPs, which are defined as metabolites secreted by organisms and their degradation products formed by biodegradation, photolysis, and/or hydrolysis, have received the most attention in terms of their properties and effects [85,86,87,88,89]. NTS workflows have effectively found the TPs of organophosphate ester from different aquatic organisms [90]. For example, in *Daphnia magna* (*D. magna*), Choi et al. (2020) [91] used NTS to identify the TPs of flame retardant triphenyl phosphate. For model organisms, various target analyses have been conducted [92,93]. However, the NTS technique has only been used in a few studies; therefore, there is a lack of knowledge on novel TPs rising concerns in model organisms.

### 3.1. Sample Treatment and QA/QC

Despite significant advances in implementing QA/QC practices for analytical processes, a substantial nontarget analysis of TPs in organism matrices poses new obstacles. Consequently, an adequate set of QA/QC techniques based on metrological traceability of findings is critical for understanding the reasons for unwanted variances and minimizing them. The analytical laboratory should provide clear criteria for the execution and assessment of each QA/QC activity, identifying the parameter under evaluation and detailing how parameters might be modified to use the set of QA/QC activities properly. The analyst should determine the parameter examined for each QA/QC operation and its excellent decision value based on the study’s unique requirements (i.e., objectives, number of samples, matrix, and predicted substances) [94,95]. While implementing all the specified QA/QC steps is not required and is dependent on the project’s objectives, implementing specific measures for each analytical stage is necessary to provide accurate findings.

### 3.2. Sample Extraction for Non-Target Screening

For tissue sample extraction, microwave-assisted extraction (MAE), ultrasonic-assisted extraction (UAE), Quick, Easy, Cheap, Effective, Rugged, and Safe (QuEChERS), and pressurized liquid extraction (PLE) have been applied in TP studies (Table S1). 

#### 3.2.1. Quick, Easy, Cheap, Effective, Rugged, and Safe

Using the QuEChERS approach, minimal solvent consumption, fast extraction, and effective clean-up can be achieved with minimized cost [96]. In addition, it is highly customizable, thus becoming a popular pretreatment method for various biota samples. Analyte characteristics, matrix composition, and analytical method influence efficacy [96]. In general, following a salting-out extraction with solvent (e.g., methanol, acetonitrile, etc.), the QuEChERS method employs a dispersive solid-phase extraction (d-SPE) to reduce matrix effects. An example is the following. Tests of QuEChERS for the extraction of diclofenac and its TPs in bivalves were conducted by Daniele et al. (2016) [97]. A 50 mL polypropylene centrifuge tube was used to weigh a 100 mg aliquot of homogenized and freeze-dried sample. Extracts of the target analytes were spiked up to 200 mg/L and diclofenac-d4 (100 mg/L) in a methanolic mixture, and the solvent was evaporated under a moderate stream of nitrogen for method development. The dry extract was mixed with 5 mL of water to aid in the salting-out extraction process. It was then mixed with 10 mL of acetonitrile (ACN) and 200 mL of heptane and vortexed for 15 s. Then, acetate salt was added, vigorously agitated for 10 s, and vortexed for 20 s. The homogenate was centrifuged for 3 min at 10,000 rpm to remove the acetate salt. The 6 mL of ACN was transferred to a 10 mL glass tube. To keep the analytes from evaporating, 200 mL of DMSO was added with a moderate stream of nitrogen used to evaporate ACN. As a final step, the remaining DMSO solution was mixed with 50 mL of ^13^C phenacetin (1 mg/L in ACN) (Figure S2). Quantitative analysis for the residue was carried out using LC–MS/MS.

#### 3.2.2. Microwave-Assisted Extraction

Two different microwave systems, i.e., closed extraction vessels/multi-mode microwave ovens and ‘open’ focused microwave ovens, have been used in the laboratory for extraction. The most often utilized systems today are closed vessel-like systems (Figure S3), in which the extraction efficiency is determined by applied temperature and pressure. MAE minimizes sample consumption and extraction time by providing a high throughput extraction. In certain circumstances, replacing the extraction phase with water instead of an organic solvent resulted in a more effective extraction [98]. The extraction efficiency was influenced by the solvent’s volume and properties, the irradiation period, and microwave power [99]. The target contaminants would decompose due to the high microwave power and lengthy irradiation [100]. Wang et al. (2012) [101] suggested a technique for determining nine steroid hormones in fish tissues based on MAE. Under the influence of microwave radiation, the hormones were extracted with acetonitrile and water. The extract was then separated with ammonium acetate into an acetonitrile phase. The target analytes in the phase were concentrated and analyzed using LC–MS/MS. The recovery values for the method were 78.9–94.3%.

#### 3.2.3. Pressurized Liquid Extraction

PLE is a simple and comprehensive extraction process that enables quantitative recoveries with minimal effort on method development. A general procedure is as follows. The cell is put on the carousel once the sample has been entered and mixed with the inert substance. The sample cell is rotated on the carousel before transferring to the oven and immediately sealed under pressure. The cell is then filled with solvent and held in the range for a user-defined time at a persistent pressure and temperature. After collecting the extracted analytes in a vial, the cell is washed and purged with nitrogen gas. These phases form a cycle. The overall extraction time is, in general, 15–45 min. PLE increases the extraction of contaminants from solid materials using standard solvents at raised pressure. It may result in a large percentage of matrix interference being extracted [102]. Pressurized hot water extraction offers an alternative as an ecologically gentle approach [103].

#### 3.2.4. Ultrasonic-Assisted Extraction

The UAE extraction process includes homogenizing, freeze-drying, sieving, centrifuging, ultrasonication, and clean-up processes [104]. UAE is a fast and efficient way to extract natural chemicals and contaminants from food and environmental samples, with extraction efficiency equivalent to traditional methods [105]. The UAE approach is a low-cost alternative to contemporary extraction procedures that are simple and may be employed with any solvent. Dorival-Garc’a et al. (2013) explored the productivities of PLE, UAE, and MAE techniques and identified that the methods had similar extraction times, with PLE and MAE providing the best extraction yield [106]. The proposed UAE is an alternative because it is simple to employ, has equal solvent volumes, and has similar precision and sensitivity. Wilkinson et al. (2007) compared the MAE and UAE methods to build a methodology for determining 13 chemicals in aquatic plants and benthic organisms. The UAE-SPE technique offered the best results [89]. Qu et al. (2017) used UAE to minimize extraction time and enhance extraction efficiency to analyze amide herbicide residues in fish [105].

## 4. Transformation Pathway in a Model Organism

In vitro and in vivo metabolic processes may transform organic pollutants into highly reactive metabolites. The principal biotransformation routes accompany reduction, oxidation, and/or hydrolysis during the phase I reaction. In contrast, the phase II reaction mainly features conjugation reactions. Phase II reactions are biosynthetic, as active enzymes connect the metabolite produced by phase I responses to an endogenous polar molecule, resulting in a conjugate. Many endogenous compounds with high polarity (e.g., sugar, amino acids, sulphates, etc.) are used in conjugation, and the resultant conjugates are mostly ionized and highly water soluble. Furthermore, specialized active transport systems identify the moieties employed for conjugation, assisting translocation across plasma membranes and increasing the excretion rate [107,108]. The endoplasmic reticulum, lipoprotein membranes stretching from mitochondria and nucleus to the plasma membranes of cells, are the primary sources of phase I enzymes in cells. As lipophilic substances preferentially diffuse into lipid membranes the presence of phase I enzymes in lipid membranes has crucial implications for biotransformation [109]. 

Phase I reactions are more typically related to the production of reactive and more hazardous metabolites; yet, phase II processes, as well as combinations of phase II and phase I processes, may be considered an intoxication procedure [110,111]. However, there is evidence that metabolites of pollutants such as tetrabromobisphenol-A, trenbolone, triclosan, and bisphenol A retain the bioactive moieties and preserve inherent toxicity comparable to the parent compound [112,113,114]. Methylation in biological systems can produce hydrophobic and bioaccumulative metabolites, often observed in fungus, plants, and bacteria [115]. Compound biotransformation studies are vital to understand the reactivity and toxicity of organisms. Bioaccumulation and toxicity of organic pollutants are heavily influenced by biotransformation, while this process is still poorly understood for emerging contaminants [116]. There have been limited investigations for TPs of EPs in specific organisms, as follows.

### 4.1. Algae

*Cymbella* sp. were studied for their ability to biotransform triclosan. The results demonstrated that triclosan and its potential hazardous metabolites had a high toxic impact on *Cymbella* sp., with 72 h EC_50_ of 324.9 mg/L. In diatom cells, 11 metabolites were found and with potential degradation pathways. The transformative reactions of triclosan in *Cymbella* sp. included methylation, hydroxylation, amino acids conjunction, dichlorination, and glucuronidation, which resulted in biologically active products (e.g., methyl triclosan) and conjugation products (e.g., or oxaloacetic acid conjugated or triclosan glucuronide) [117].

### 4.2. Freshwater Crustaceans

**Figure 4:**
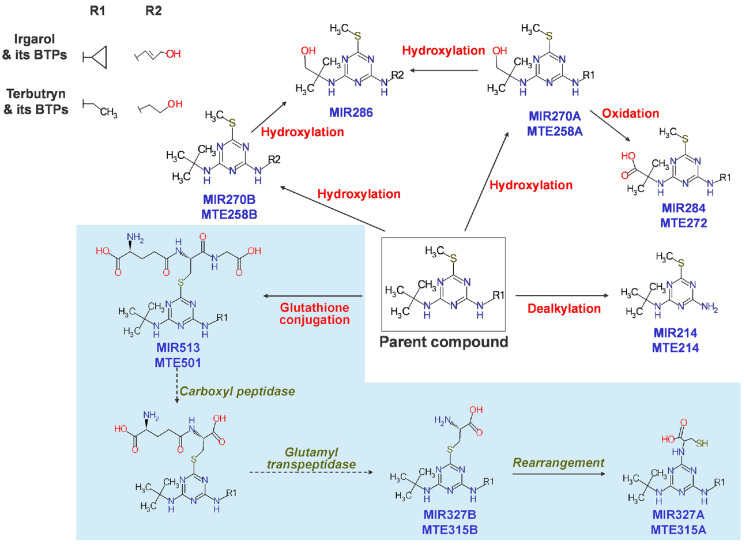
Proposed biotransformation pathways of irgarol and terbutryn in freshwater crustaceans. Note that R2 is the hydroxylated moiety of R1. The sky-blue shaded area indicates a pathway including glutathione conjugation followed by subsequent reactions to form cysteine conjugates, reported for the first time in the test organisms. (Reprinted from [118], Copyright 2013, ACS).

Biotransformation pathways in freshwater crustaceans have been little understood, except in a study using *Gammarus pulex* (*G. pulex*) and *Daphnia magna* (*D. magna*). For 24 h, *G. pulex* and *D. magna* were exposed to a modest dose of biocides and pharmaceuticals and sacrificed to identify their metabolites. Each species produced 25 and 11 metabolites, respectively, for terbutryn, irgarol, venlafaxine, and tramadol, mainly via oxidation and conjugation reactions. Affinity in the synthesis of metabolites, such as oxidation and demethylation products, were found for venlafaxine and tramadol, which have an identical backbone structure. Tramadol and venlafaxine were oxidized at the amine or cyclic C-H bond, while irgarol and terbutryn were oxidized at the terminal methyl group (MTE258B, MIR270B, MIR270A, MTE258A, and MIR286) (Figure 4) [118]. In *Gammarus pulex* (*G. pulex*) and *Hyalella azteca* (*H. azteca*), Fu et al. (2020) found a substantial pathway for diclofenac metabolism [119]. The LC–HRMS/MS data collected from the test species were used to identify metabolites utilizing NTS procedures. As a result, 281 metabolites were identified based on the isotopic signature of chlorine (Cl). *H. azteca* and *G. pulex* had nine distinct diclofenac metabolites.

### 4.3. Fish

Metabolites of diclofenac were also identified in vertebrates. *Oncorhynchus mykiss*, have produced hydroxylate and conjugate of diclofenac with glucuronic acid, glutathione, and sulfate. Other EPs, including pharmaceuticals (propranolol, carbamazepine) and insecticides (diazinon, azoxystrobin, and fipronil), were found in the S9 extract of trout liver [120]. It was revealed that each of the five parents had ten distinct metabolites. The primary metabolic mechanisms were oxidation, dealkylation, S-oxidation, and epoxidation. The formation of metabolites for fipronil and diazinon was enhanced as increasing carbamazepine concentration in the binary exposure, whereas the transformation kinetic for propranolol and azoxystrobin was decreased. Toxic diazoxon and less toxic pyrimidinol, among significant diazinon metabolites, were promptly formed by S9 after the binary exposure with carbamazepine. 

### 4.4. Earthworm

High-production-volume surfactants, also known as polyfluoroalkyl phosphate esters (PAPs), are employed in the packaging industry and food contact paper. PAPs can transform into perfluoroalkyl carboxylic acids, which are highly bioaccumulative and persistent in the environment, although their fate remains unknown in terrestrial species. To investigate biotransformation, Zhu et al. (2021) subjected *M. guillelmi* to soil contaminated with 6:2 fluorotelomer phosphate diester (6:2 diPAP). According to in vitro desorption tests [121], 6:2 diPAP desorbed from soil was considerably accumulated in gut digesting fluid. Phase I products included perfluoropentyl propanoic acid, perfluorohexanoic acid, 2-perfluorohexyl ethanoic acid, perfluoropentanoic acid, and perfluoroheptanoic acid, all of which confirmed that β and α oxidation occurred in earthworms. As a phase II product, 6:2 fluorotelomer alcohol–sulfate conjugate was found at unusually high quantities in earthworms for the first time, which may be the principal mechanism by which earthworms remove 6:2 diPAP.

### 4.5. Human Cell Lines

Using human skin subcellular fractions, the in vitro metabolism of 2-ethylhexyl-2,3,4,5-tetrabromobenzoate (EH-TBB) and a mixture of Bis(2-ethylhexyl) tetrabromphthalate (BEH-TEBP), EH-TBB, and triphenyl phosphate was evaluated for the first time. Analysis of EH-TBB and THP utilizing UPLC-Q-Exactive Orbitrap identified the two primary metabolites, tetrabromobenzoic acid (TBBA) and diphenylphosphate (DPhP). It was assumed that CYP450 enzymes were responsible for the dermal biotransformation of TPhP and EH-TBB, but no stable metabolites were found for BEH-TEBP [122].

## 5. Toxicity Effect of TPs on the Model Organism

### 5.1. Potential Bioactivation Mechanisms

**Figure 5:**
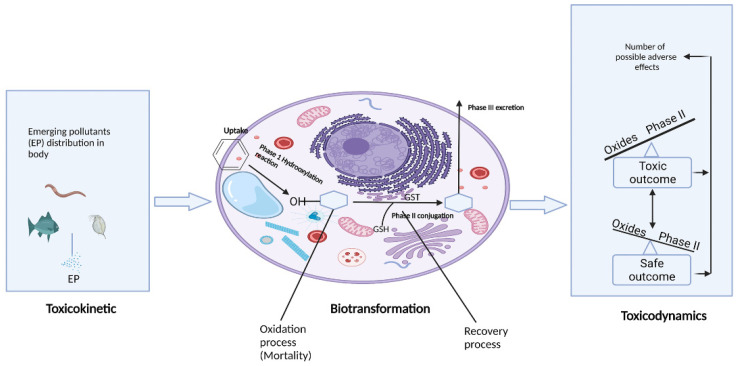
Schematic biotransformation processes result either in toxic or safe products.

Biotransformation may undergo a two-step process. In the first phase, including reduction, oxidation, and/or hydrolysis, the enzymes interact with the EPs and convert them into water-soluble metabolites, in many cases facilitating excretion from the body [123,124,125]. The biotransformation process generates a variety of intermediate and final metabolites that might result in any of the following outcomes: toxicity, inertness in the body, or safe elimination [125]. Cytochrome P 450 group enzymes CYP1 are active in the first step of EPs biotransformation [123]. There must be an equilibrium between the generation of oxygen atoms and other natural processes such as biotransformation to avoid any unwanted consequences. In phase II processes, metabolites generated in phase I become more water-soluble. Glutathione-S-transferase aids in phase II conjugation processes with glutathione (GST). Antioxidant protection is provided by this enzymatic activity [126]. Phase I metabolites may induce cytotoxicity if they do not undergo the phase II procedure. Due to biotransformation, cell death is caused when the hazardous compounds produced in phase I surpass the organism’s ability to conjugate them via phase II (Figure 5) [127]. The CYP1A enzymes produce a majority of the biotransformation products. Figure 5 shows the balance of activities of phase I and phase II enzymes, which define the type and effects of the metabolites generated in the biotransformation process, as the process of detoxification or toxification is depicted.

### 5.2. Cases for the Formation of Toxic TP

TPs formed by photoinduced degradation of carbamazepine (e.g., acridine, acridone) have been evaluated for toxicity [128]. Toxic effects reached their maximum for *Pseudokirchneriella subcapitata* (*P. subcapitata*) and *Vibrio fischeri* (*V. fischeri*). Exposure of *D. magna* to mixed TPs resulted in immobility after 45 min. Based on estimated EC_50_, NOEC, and LOEC, the EC_50_ for *D. magna* was 0.71 mg/L for acridine and 1.49 mg/L acridone, but the NOEC value for carbamazepine was 30 mg/L for this species. Acridine was found to be the most hazardous to algae (EC_50_ = 0.61 mg/L) and *V. fischeri* (EC_50_ = 5.34–6.90 mg/L) in the experiments [128]. Grabarczyk et al. (2020) also found that carbamazepine-10,11-epoxide was still harmful to diverse organisms compared to the parent. At 100 mg/L, the luminescence inhibition rate of *V. fischeri* by Carbamazepine-10,11-epoxide was 30%, whereas the effect was 42% for carbamazepine. *Lemna minor* (*L. minor*) showed similar responses to the epoxide TP and the parent at 50 mg/L (the epoxide inhibits growth by 32% and carbamazepine by 49%). Both were critical to the growth of green algae (48 %). Similar inhibitions were observed for *R. subcapitata* (15 %) and *L. minor* (11%) exposed to 10,11-dihydro-10-hydroxycarbamazepine. On the other hand, for *D. magna*, both metabolites of carbamazepine were ineffective, while 24% of the exposed species to the parent was found to be immobilized [92]. 

Boillot et al. (2015) investigated the internal concentrations of carbamazepine and oxcarbazepine-10-hydroxy-10,11-dihydrocarbamazepine in biota. After the exposure of *Mytilus galloprovincialis* to the chemicals for a week, concentrations in media and biota were measured and evaluated for bioconcentration. According to the evaluation, 10-hydroxy-10,11-dihydrocarbamazepine showed slightly higher bioconcentration factors than carbamazepine. Carbamazepine-10,11-epoxide and acridine accumulated in the mussel following the exposure. Both chemicals were found in the digestive glands and gills. Acridine was also detected in mantels and measured in digestive glands. This might imply that metabolites can be accumulated in organisms due to direct uptake of the chemical from the media and/or as a resultant of metabolization from the parent [129].

Similarly, carbamazepine was found in fish after 48 h of exposure in the study of Valdés et al. (2016) [130]. The gills and muscle of *J. multidentata* contained 2-hydroxycarbamazepine (48 to 107 × 10^−6^ mg/g wet weight), while the epoxide TP (41 × 10^−6^ and 60 × 10^−6^ mg/g wet weight) was also found. It is indicated that exposed organisms biotransform carbamazepine and accumulate at similar concentrations. Another study provided information on the TPs of β- blockers and their toxicity. The harmful effects of propranolol investigated on *B. calyciflorus* and *P. subcapitata* exposed at concentrations from 1 and 10 mg/L. The findings for both species demonstrated that the irradiated sample had a greater EC_50_ value (lower toxicity) than the parent compounds at each stage of the experiment (6, 24, 48 h). During photolysis, the toxicity was decreased by 1.8 to 3 times [131]. The opposite findings were observed in research of photodegradation products of metoprolol and atenolol [132]. Their photo-TPs had a greater level of toxicity than the parent compound. A significant increase in luminescence inhibition of *V. fischeri* was seen after 16 and 32 min of metoprolol and atenolol irradiation (Toolaram et al. (2017)).

## 6. Challenges and Perspectives for TP Study

The TPs relevant literature has demonstrated a growing requirement for realistic methodologies to examine the ecotoxicity of TPs. More research is required on the effects, interactions, uptake, and translocation of TPs in organisms due to the rising manufacture of pharmaceuticals, insecticides, and industrial chemicals (e.g., organophosphate flame retardants). One of the main focuses of this review is on how NTS has been used in the model organism, TPs pathway, and toxicity effects. In terms of the ecological consequences of TPs, a number of studies revealed a wide range of outcomes showing increased or reduced toxicity on model species. TP formations in biota are primary consequences of phase I and phase II reactions after the parent molecule is introduced into the organism. At this step, the parent chemical goes through numerous phases of oxidation, hydrolysis, and enzymatic reaction, all of which might influence the TP structure and toxicity. The transformation route has been found to enhance or reduce ROS production, survival rate, and bioavailability of TPs in model organisms in previous research.

Furthermore, the hypothesized route has yet to be defined as having a direct influence on the toxicological effects of TPs. Thus, more research should be carried out to determine the impact of TPs. A reliable model must be developed to define and assess TPs with a live organism. As a prerequisite, it is critical to identify as many TPs as possible with reliable information on the chemical structures. Currently, most research relies on target screening for identification, while a few studies have been performed on the unknown TPs using NTS in the absence of reference standards. Even though the identification confidence is not always reliable, efforts and trials to find novel TPs must be continued. As a helpful tool, the software-aid approach can predict the transformation products and pathways [124,125,126]. In parallel, ecotoxicology investigations should also include NTS for their potential significance in the characterization of unknown TPs and their toxicological consequences.

HRMS, combined with advanced hardware and software, has helped detect unknown migrants from emerging contaminants. Since ESI is the most extensively utilized ionization source, a significant trend toward using HRMS in LC for non-targeted analysis has been noticed in recent years. The developing field of NTS is characterized by both a very complicated scientific setting and a fundamental methodological framework. Each stage of the process in this discipline presents considerable obstacles requiring analytical improvement. Tissue samples need different methods and procedures for other environmental samples such as water and sediments. Efforts to obtain improved consolidation and comparability of data gathered from multiple studies, particularly for use in regulatory and policy support contexts, need a strict harmonization approach. However, to sustain its potential for discovery and exploratory study, this expanding area needs a high degree of adaptability. A combination of LC-HRMS and GC-HRMS is necessary to cover many EPs and their numerous TPs, requiring high-level equipment and extensive technical competence. 

On the other hand, to handle the complexity of NTS data, it is essential to use cutting-edge computational tools, many of which are still in development. Furthermore, analytical techniques combined with informatic skills must be practically implemented to expand knowledge on TPs and characterize both TPs and the parent pollutants.

## Figures and Tables

**Table 1:** Pharmaceuticals and metabolites/TPs detected in Effulent waste water and surface water samples after retrospective search in Q-TOF- MS data. (Reprinted from [46]. (Copyright 2016, Elsevier)).

	Positive Finding (%)	
	EWW (*n* = 38)	SW (*n* = 18)
Irbesartan	92	39
ISW1	87	6
IB3	84	22
IB3	89	22
IB4	32	11
IB5	79	22
Valsartan	79	33
Venlafaxine	87	22
VB1a	92	17
VB1b	92	17
V1	58	6
V2	87	11
Ofloxacin	82	17
Ibuprofen	11	6
IbSW2a	16	11
IbSW2b	8	0
IbB4	34	50
Ib1	21	6
Gemfibrozil	24	22
GSWB1	71	33

**Table 2:** Possible biological and chemical transformation reactions and associated mass changes. (Reprinted from [82], Copyright 2021, Elsevier).

Transformation	Change in Molecular Formula	Mass Change (Da)	Parent Chemical	Parent Structure	Product Structure	Reference
loss of nitro group	–NO_2_ + H	−44.9851	Chloramphenicol	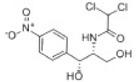	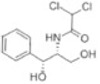	[65]
decarboxylation	–CO_2_	−43.9898	Ibuprofen	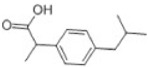	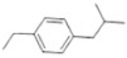	[66]
depropylation	–C_3_H_6_	−42.0468	Ibuprofen	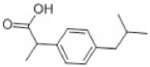	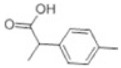	[66]
reductive displacement of chlorine	–Cl + H	−33.9611	Triclocarban	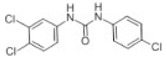	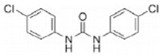	[67]
desethylation	–C_2_H_4_	−28.0312	Lidocaine	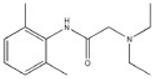	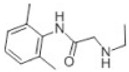	[68]
dehydration	–H_2_O	−18.0106	Hydroxylation ibuprofen	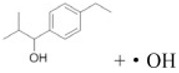	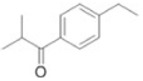	[69]
reductive displacement of fluorine	–F + H	−17.9906	Ciprofloxacin	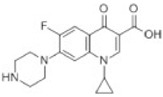	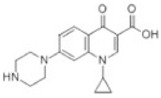	[70]
oxidative displacement of chlorine	–Cl + OH	−17.9662	Ceftriaxone	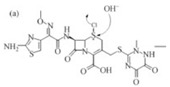	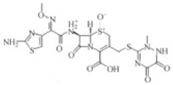	[71]
demethylation	–CH_2_	−14.0157	Naproxen	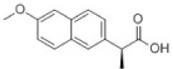	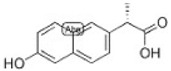	[72]
hydrogenation reduction	0	+2.0157	Carbamazepine	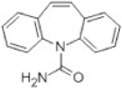	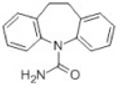	[73]
alcohol to carboxylic acid	−2H + O	+13.9792	O-desmethylmetoprolol	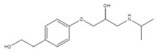	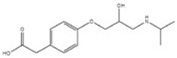	[59]
ketone/aldehyde formation	−2H + O	+13.9792	2,6-di-tert-butyl-4-methylphenol	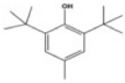	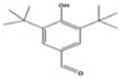	[74]
N/S-oxidation	+O	+15.9949	2-Acetylaminofluorene	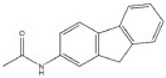	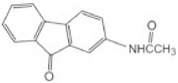	[75]
epoxidation	+O	+15.9949	Carbamazepine	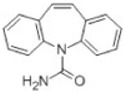	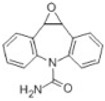	[73]
hydroxylation	+O	+15.9949	Diclofenac	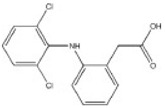	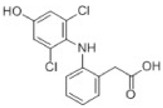	[72]
hydration	+H_2_O	+18.0106	Carbamazepine	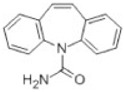	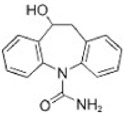	[73]
methyl to carboxylic acid	–2H + O_2_	+29.9741	Δ-9-tetrahydrocannabinol	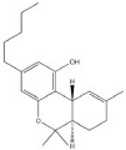	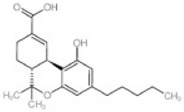	[76]
dihydroxylation	0	+31.9898	Carbamazepine	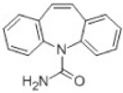	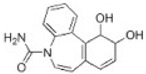	[73]
chlorination	+Cl-H	33.9612	Methylparaben	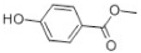	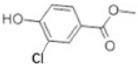	[77]
dichlorination	+2Cl	69.9173	Acetaminophen	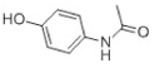	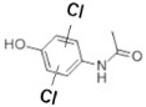	[78]
acetylation	+C_2_H_2_O	+42.0106	Sulfamethazine	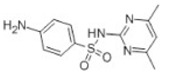	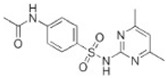	[79]
hydroxyl chlorination	+Cl + OH	51.9718	Carbamazepine	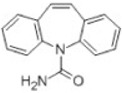	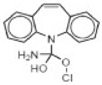	[78]
oxidative deamination	−NH + O	+ 0.9840	Amphetamine	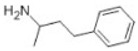	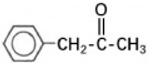	[80]
methylation	0	+14.0157	Thiouracil	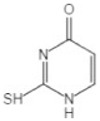	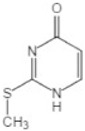	[80]
sulfate conjugation	0	+79.9568	Triclosan	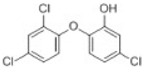	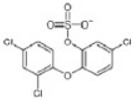	[67]
glycine conjugation	+C_2_H_3_NO	+57.0215	Benzoic acid	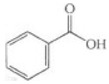	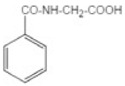	[81]
taurine conjugation	+C_2_H_5_NO_2_S	+107.0041	Bile acids	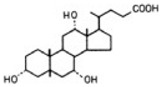	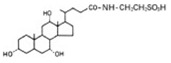	[80]
S-cysteine conjugation	+C_3_H_5_NO_2_S	+119.0041	2-acetomido-4-chloromethylthiazole	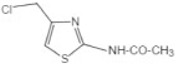	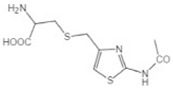	[80]
N-acetylcysteine conjugation	+C_6_H_8_NO_3_S	+161.0147	Naphthalene	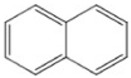	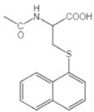	[80]
glucuronide conjugation	+C_6_H_8_O_6_	+176.0321	Testosterone	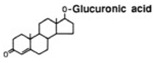	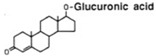	[80]
S-glutathione conjugation	+C_10_H_15_N_3_O_6_S	+305.0682	Oxidation phenacetin	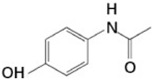	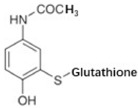	[81]

## Data Availability

As this a review article, the data supporting the results can be found in the respective references in the manuscript.

## Supplementary Materials

The following are available online at https://www.mdpi.com/article/10.3390/toxics10020054/s1, Figure S1: The contaminants selected and their metabolites/TPs in different EWW samples; Figure S2: The diagram of separation process of diclofenac and transformation product; Figure S3: (A) Schematic view for a closed-vessel MAE system and (B) Commercial MAE instrument from Milestone and Carousel with 40 positions; Table S1 Summary for sample extraction method.
