# Supplementary material for: Transformation Products of Emerging Pollutants Explored Using Non-Target Screening: Perspective in the Transformation Pathway and Toxicity Mechanism—A Review
Source: Toxics. 2022 Jan 24;10(2):54. doi: 10.3390/toxics10020054 (PMC8874687; doi:10.3390/toxics10020054)
Supplement: Supplementary file 1 [file toxics-10-00054-s001.zip › toxics-1552078 proof supplementary.pdf]

toxics

MDPI

Supplementary Materials: Transformation Products of Emerging Pollutants Explored Using Non-target Screening: Perspective in the Transformation Pathway and Toxicity Mechanism—A Review.

Thodhal-Yoganandham Suman, Soo-Yeon Kim, Dong-Hyuk Yeom and Junho Jeon

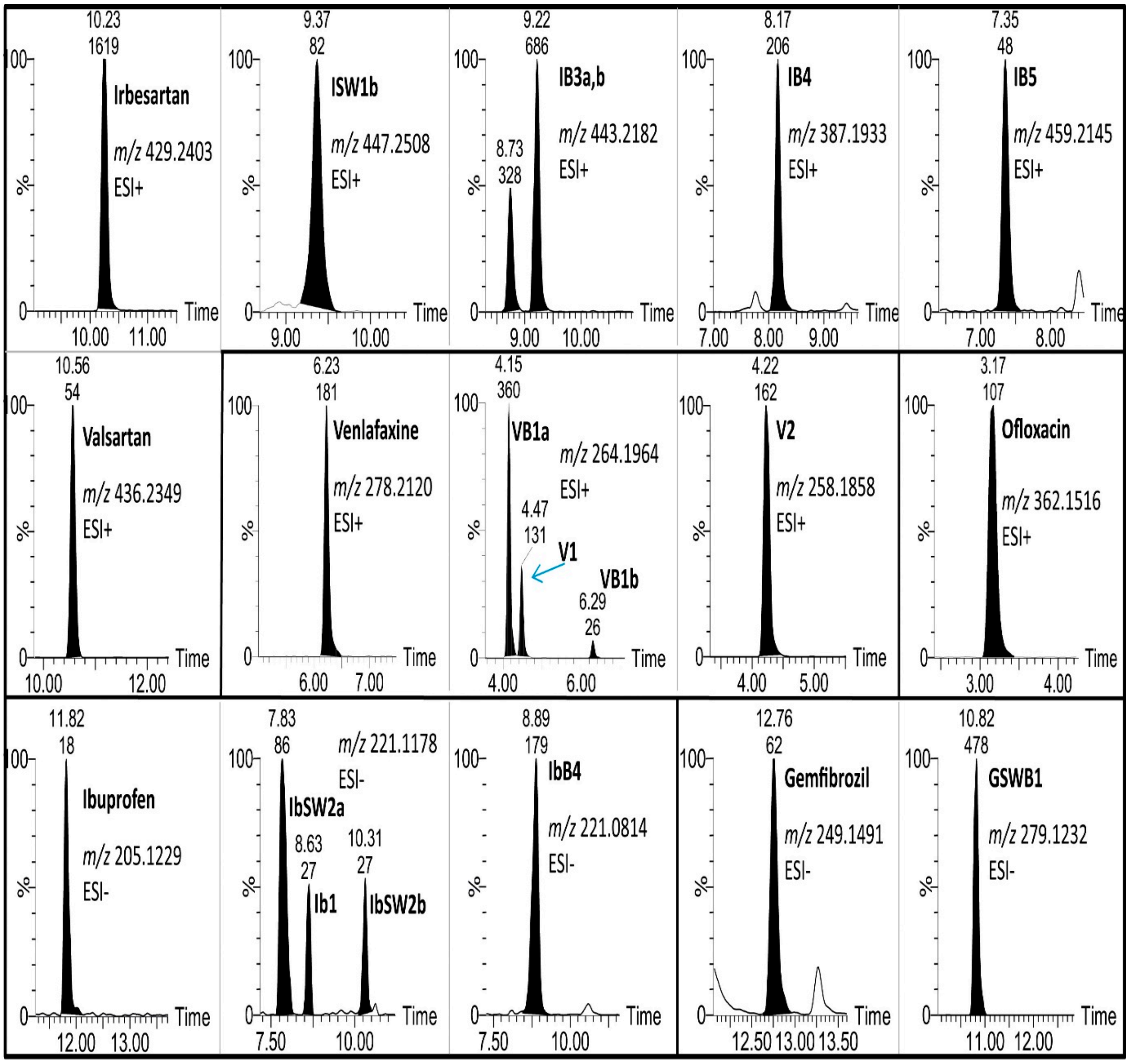

Figure S1. The contaminants selected and their metabolites/TPs in different EWW samples. (Reprinted from [46]. (Copyright 2016, Elsevier).

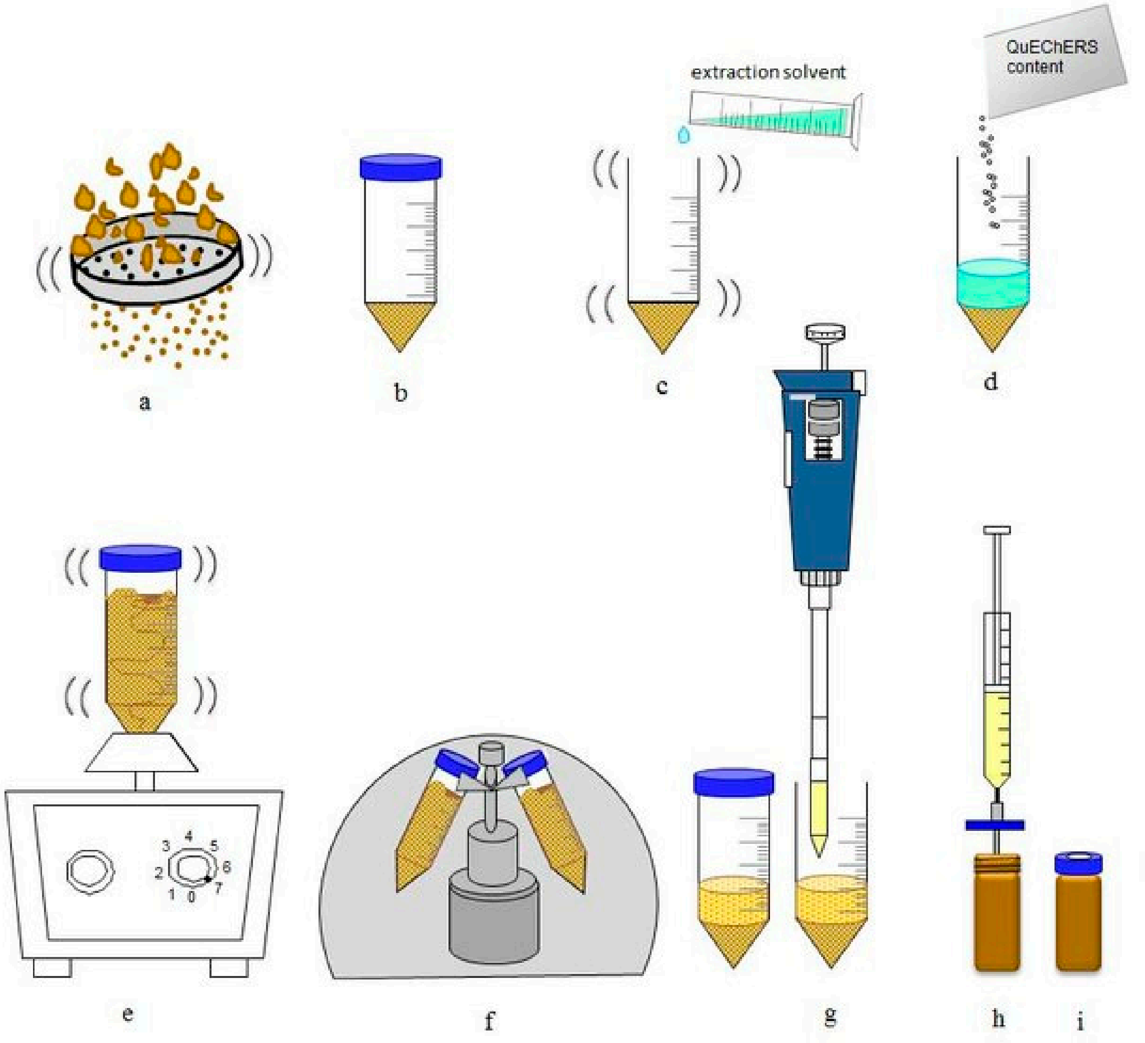

Figure S2. The diagram of separation process of diclofenac and transformation product. (Reprinted from [1], with permission of the publisher).

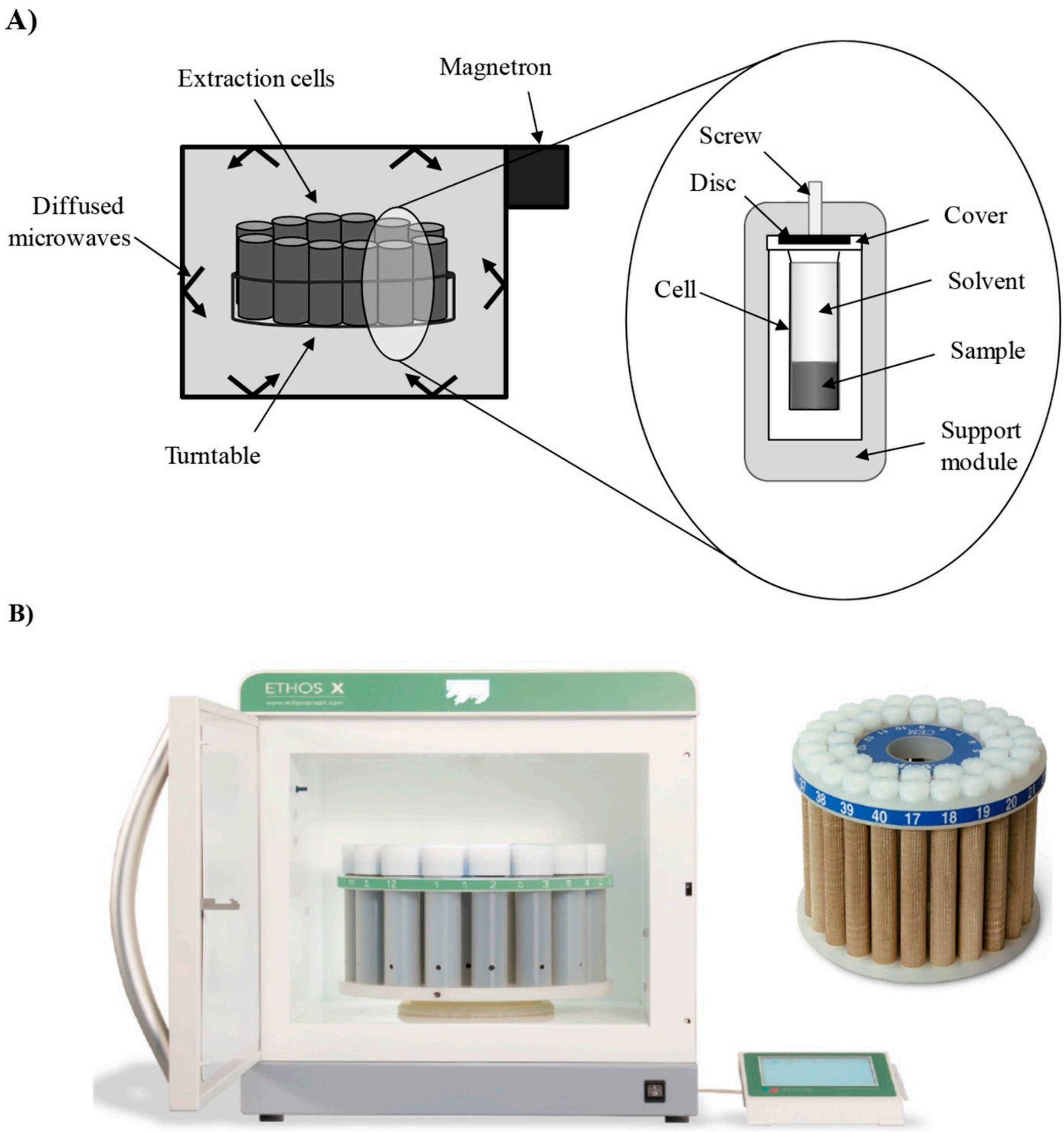

Figure S3. (**A**) Schematic view for a closed-vessel MAE system and (**B**) Commercial MAE instrument from Milestone and Carousel with 40 positions. (Reproduced with permission of Milestone, Inc.). (Reprinted from [2]. (Copyright 2019, Elsevier)).

Table S1 Summary for sample extraction method

Type of extraction	Principle	Advantages	Disadvantages
QuEChERS	The principle is similar solid phase and high performance liquid chromatography extraction. To achieve impurity removal, the adsorbent filler interacts with contaminants in the matrix to absorb impurities.	Short Method development time<br>Low Cost	Large volumes of organics<br>Difficult to automate
Microwave-assisted extraction	Small amount of solvent (15–40 mL), Fast extraction (12–18 min) ,Automated Easy to use Filtration not required, Large amount of sample (up to 100 g)	The reduction of the extraction time and solvent consumption	Clean-up required<br>Expensive equipment
Pressurized liquid extraction	Pressurized liquid extraction also known as pressurized fluid extraction or accelerated solvent extraction, is a method of extracting a sample using conventional solvents under high pressure and temperature.	Less extraction time and Closed system	An further drawback of PLE is its inability to effectively extract analytes from water-rich samples when utilizing hydrophobic organic solvents.
Ultrasonic-assisted extraction	Ultrasonication is the act of applying frequencies to agitate particles in a sample for extraction of analyte from tissue sample	speed dissolution, by breaking intermolecular interactions.	More expensive than other methods.

References

1) Vera, J., Correia-Sá, L., Paíga, P., Bragança, I., Fernandes, V.C., Domingues, V.F. and Delerue-Matos, C., 2013. QuEChERS and soil analysis. An Overview. *Sample Preparation*. **2013,** *1*, 54-77.
2) Llompart, M., Celeiro, M. and Dagnac, T., 2019. Microwave-assisted extraction of pharmaceuticals, personal care products and industrial contaminants in the environment. *TrAC Trends in Analytical Chemistry*, **2019**, *116*, 136-150.
